# Supplementary material for: Dominance between self-incompatibility alleles determines the mating system of Capsella allopolyploids
Source: Evol Lett. 2024 Mar 17;8(4):550–60. doi: 10.1093/evlett/qrae011 (PMC11291619; doi:10.1093/evlett/qrae011)

Dominance between self-incompatibility alleles determines the mating system of Capsella allopolyploids

Tianlin Duan, Zebin Zhang, Mathieu Genete, Céline Poux, Adrien Sicard, Martin Lascoux, Vincent Castric & Xavier Vekemans

Supplementary information

Analysis of *S*-locus genotypes and quantification of relative transcript abundances using the bioinformatic pipeline NGSgenotyp

Identification of S-alleles and classification into four dominance classes

Despite the fact that most *S*-alleles are shared transpecifically between *A. halleri* and *A. lyrata* (Castric et al. 2008) and even transgenerically between *Arabidopsis* and *Capsella* (Paetsch et al. 2006), *S*-allele identifications/names have been assigned independently in each species, leading to much confusion in the literature. In this paper we introduce an identification scheme based on phylogenetic relationships among *SRK* sequences, in the form HXYYY, with X corresponding to the dominance class of the allele (ranging from 1 to 4), and YYY corresponding to its functional specificity (ranging from 001 up to a limit of 999). The functional specificity of *SRK* sequences from different species is assumed to be shared if they are phylogenetically closer to each other than to any other sequence from the same species (Castric et al. 2008). For instance, the most recessive allele in Arabidopsis and Capsella, called AhSRK01 in *A. halleri*, AlSRK01 in *A. lyrata* and CgrSRK03 in *C. grandiflora*, is here noted H1001 because it belongs to the dominance class I and is the first and only specificity in this class. The equivalence between species-specific identifications and our new generalized identification system is listed in Table S2. The dominance classes are also defined based on phylogenetic relationships among *SRK* sequences, as suggested initially by Prigoda et al. (2005) who defined four classes, as follows from the most recessive to the most dominant: A1, B, A3, A2. However, for more clarity and simplicity, we use instead the notation introduced by Goubet et al. (2012), from I to IV in the direction of increase in dominance: I (A1), II (B), III (A3) and IV (A2).

Reference SRK and SCR sequences from Arabidopsis and Capsella

Obtaining *S*-locus genotypes using the NGSgenotyp pipeline (Genete et al. 2020) requires a series of reference *SRK* and/or *SCR* sequences, so we started by assembling them from the literature. For *A. halleri*, the reference *SRK* sequences were taken from Table S1 of Genete et al. (2020). For *A. lyrata* they were taken from Table S14 of Mable et al. (2018) and from Takou et al. (2021). For *C. grandiflora* we used a set of partial *S*-domain sequences obtained by Paetsch et al. (2006), Guo et al. 2009, Bachmann et al. 2019, Nasrallah et al. 2007 and Neuffer et al. 2023. Reference sequences of *SCR* from *A. halleri* and *A. lyrata* were taken from Guo et al. (2011), Goubet et al. (2012), and Durand et al. (2014). All *SRK* and *SCR* reference sequences used are posted at https://www.doi.org/10.6084/m9.figshare.22567558.v2 together with their Genbank accession numbers.

S-locus genotypes and SRK sequences of the Cg-9 population of C. grandiflora

We expanded the reference database of *Capsella* reference *SRK* sequences by using the *de novo* assembly module (haploasm) of the NGSgenotyp bioinformatic pipeline (Genete et al. 2020) based on Illumina short-read whole genome resequencing data of 180 individuals from the Cg-9 population of *C. grandiflora* from Monodendri, Greece (Josephs et al. 2015; Sequence Read Archive (SRA) BioProject PRJNA275635, Table S1). This allowed us to first obtain full sequences of exon 1 of *SRK* for the *C. grandiflora* for which only partial sequences were available (49 in total), as well as to obtain full sequences of exon 1 of *SRK* for the newly identified alleles (25 in total). The sequences of these 74 *SRK* alleles are available at https://www.doi.org/10.6084/m9.figshare.22567558.v2. Based on this updated database of reference sequences, we then launched a new round of the genotyping module (genotyp) to obtain *SRK* genotypes. We obtained fully resolved diploid *S*-locus genotypes for 177 individuals (Table

S1), while sequence read depth was too low for two additional individuals. One individual was missing a single allele, and one individual had four alleles, which could correspond to an admixed sample or an autotetraploid individual.
Beside these 74 *S*-alleles, we also identified five sequences clustering with class II *SRK* alleles (H0002, H0003, H0011, H0012 and H0013 in Table S2, named, respectively, CgrSRK01, CgrSRK06, CgrSRK09, CgrSRK51 and CgrSRK63 in Paetsch et al. 2006 and Neuffer et al. 2023) that we considered as paralogous sequences unlinked to the *S*-locus for the following reasons : (1) two of them (H0002 and H0003) showed strong sequence similarity with sequences for which paralogy was confirmed with segregation analyses in *A. lyrata* and *A. halleri* (Aly13-2 and Aly13-7; Charlesworth et al., 2003; Castric & Vekemans, 2007); (2) the remaining three sequences had higher population frequencies than the other class II *SRK* alleles (11, 23 and 12 copies for H0011, H0012 and H0013, respectively, while true class II *SRK* alleles had frequencies ranging from 1 to 10 copies); and (3) they systematically occurred in individuals which already carried two other unambiguous *SRK* alleles (see Table S1).
Among the 74 *S*-alleles co-segregating in this population, one belongs to the most recessive class, 16 alleles belong to the second most recessive class (class II), 20 alleles belong to the more dominant class III and 37 alleles belong to the most dominant class IV (Table below). As theoretically expected (Schierup et al. 1997; Billiard et al. 2007), we observed that the more dominant classes contain a larger number of different *S*-alleles, each at a lower population frequency .

Number and mean population frequency of *S*-alleles found in the population Cg-9 of *Capsella grandiflora* belonging to each of the four classes of dominance

	Class I	Class II	Class III	Class IV	Total
Number of *S*-alleles	1	16	20	37	74
Average frequency ± S.D.	0.2436	0.0133 ± 0.0075	0.0115 ± 0.0065	0.0085 ± 0.0056	/

Validation of the NGSgenotyp pipeline for use on RNA-seq data
We evaluated how the NGSgenotyp performed when applied to RNA-seq data (from either flower bud, leaf or root tissues) by comparing the results with those obtained from whole genome resequencing data. We performed this comparison on four *C. grandiflora* and four *C. orientalis* individuals, as well as on 16 *C. bursa-pastoris* individuals from Kryvokhyzha et al. (2019, Table S1), for which both types of sequence data were available. We used as reference database of *SRK* sequences a combination of sequences from *A. halleri*, *A. lyrata*, and our enlarged set of *C. grandiflora* sequences.
For *C. grandiflora*, all four individuals were heterozygous at the *S*-locus, with allele H1001 shared by three individuals, while all other gene copies belonged to distinct *S*-alleles (H2002, H2006, H2017, H3014, and H4021) (Table S3). In addition, two individuals carried one copy each of putative paralogous sequences (H0011 and H0003 in individuals 85.3 and 86.12, respectively). For *C. orientalis*, all 4 individuals were homozygous for the non-functional allele H4004*n* (*n* is for non-functional), in agreement with Bachmann *et al.* (2019, note that these authors refer to this allele as CoS12). All *C. orientalis* individuals were also homozygous for an unlinked paralogous sequence (H0014, named CorSRK06 by Neuffer et al. 2023). Unlike the putative paralogous sequences observed in *C. grandiflora*, which are segregating at low or moderate frequencies at unlinked loci, the H0014 sequence appears to be fixed in *C. orientalis*. For the allotetraploid *C. bursa-pastoris*, in agreement with the results of Bachmann *et al.* (2021), all individuals but two had two copies of the H4004*n* allele, derived from the non-functional *C.*

orientalis parental allele, and all individuals had two copies of the H4047*n* allele, derived from the functional *C. grandiflora* parental allele H4047 (Table S3). Determining the exact copy number of alleles is possible using this dataset because analyses are based on genomic data, so that relative read depth informs about copy number (Genete et al. 2020). Indeed, as previously observed by Bachmann *et al.* (2021), accession DUB-RUS9 was lacking the H4004n allele inherited from *C. orientalis*, but we found that this individual was carrying two copies of a distinct allele, H2002, in the *C. orientalis* subgenome. Interestingly, another accession also carried one copy of the H2002 allele, LAB-RUS-4, but this accession also carried one copy of the *C. orientalis* H4004*n* and two copies of the *C. grandiflora* H4047 alleles. Hence, it appears that Russian accessions of *C. bursa-pastoris* have a second allele segregating at the *S*-locus of the *C. orientalis* subgenome, which was not reported by Bachmann et al. (2021). In addition, we found that all but five *C. bursa-pastoris* individuals have the *C. orientalis* subgenome fixed for the H0014 paralogous sequence, and all but two individuals were fixed, probably in the *C. grandiflora* subgenome, for the H0003 paralogous sequence (Table S3). The genomic locations of H0014 and H0003 were confirmed to be distinct from the *S*-locus region thanks to Blast analysis against a public genome assembly of *C. bursa-pastoris* (Kasianov et al. 2017).
Analysis of RNA-seq data from flower buds from the same set of individuals gave strictly identical results as those obtained from genomic data, confirming the codominant expression of *SRK* transcripts (Burghgraeve et al. 2020). As expected also, RNA-seq data detected no expression of *SRK* from leaf or root tissues, with only three exceptions: two *S*-alleles from class II (H2002 and H2006), and one *S*-allele from class III (H3014). The expression of *SRK* in leaf tissues was already reported for some *SRK* alleles in *A. lyrata*, all from class II (Prigoda et al. 2005). Overall, these results confirm that the NGSgenotyp pipeline can be used to reliably infer *S*-locus genotype from RNA-seq data from flower buds.

Determining patterns of allelic expression in SRK and SCR in diploid and tetraploid parents.
We combined RNA-seq data from seven diploid and six synthetic tetraploid individuals of *C. grandiflora* used as parents for the hybrid material with *C. orientalis* (Duan et al. 2023, BioProject PRJNA848625) with newly obtained RNA-seq data from additional hybrid individuals (BioProject PRJNA946929) and applied the NGSgenotyp pipeline to obtain their *S*-locus genotype by focusing first on *SRK*. Overall, five different *S*-alleles were segregating among the 13 individuals: H1001, H2008, H2022, H4015 and H4035 (Fig. 2). All these alleles were present in the Cg-9 population and thus their sequences were present in the database. We used the "average read depth" computed by the genotyping module of NGSgenotyp to quantify relative expression of the *SRK* alleles present within each diploid and tetraploid individual. The results showed overall co-dominant transcript levels of the different *SRK* alleles (Table S4). Note that in the tetraploid individuals analyzed, we observed up to only three different *SRK* alleles, which implies that one of the three is present in two copies. However, the use of RNA-seq data precludes reliable determination of *SRK* copy numbers, as can be done using genomic resequencing data (see above).
Then we aimed to obtain the associated *SCR* sequences, so applied again the *de novo* assembly module (haploasm) of NGSgenotyp on the same individuals, but this time using the library of *SCR* reference sequences from *A. halleri* and *A. lyrata* described above, and using shorter k-mers (length of 15 instead of the default value of 20). Although reference *SCR* sequences were available only for H1001 and H2008 (from *A. lyrata*), we successfully obtained full coding sequences for all five *S*-alleles. The sequences are available at https://www.doi.org/10.6084/m9.figshare.22567558.v2. We used the *SCR* reference sequence database enriched with these five newly obtained *SCR* sequences to launch a new round of the genotyping module (genotyp) to estimate the relative expression of *SCR* alleles. In the reference database we removed exon 1 sequences of each SCR allele because they show lower allelic specificity which brings noise in the analyses. As the *SCR* sequences from the database are small, the alignment mode was set to "local" to allow partial mapping of the reads (soft clipping) in a way that optimizes alignment score. The results showed strongly asymmetric allele-specific expressions (see main text and Table S4). The results also showed that only one allele was highly expressed in tetraploid individuals, suggesting that the small RNA-mediated regulation of *SCR* expression is fully functional in tetraploids. These results also allowed to determine the patterns of hierarchical dominance among these five *S*-alleles, as follows: (1) H4035

dominant over H4015 (individual cg4-1-3-F); (2) H4035 dominant over H2022 and H2008 (cg4-8-3-F, cg4-12-4-F, cg4-9-2-F); (3) H4015 dominant over H2022 and H2001 (cg2-1-2-F, cg2-2-6-F, cg2-1-6-F, cg2-7-3-F); (4) H2022 dominant over H2008 (cg2-12-3-F, cg2-14-5-F, cg4-6-4-F). Hence, assuming transitivity of the dominance relationships: H4035 > H4015 > H2022 > H2008 > H1001.

Determining patterns of allelic expression in SRK and SCR in diploid and tetraploid hybrids
Finally, we applied the genotyp module of NGSgenotyp with *SRK* and *SCR* reference sequences, separately, on RNA-seq data from 27 diploid *C. orientalis* x *C. grandiflora* hybrids (F), seven tetraploidized hybrids (Sh) as well as 19 autotetraploid hybrids (Sd) obtained by crossing the parents described above (Fig. 1, Table 1). Overall, five different *S*-alleles were segregating among these hybrid individuals: the non-functional *C. orientalis* H4004n allele along with four *C. grandiflora* alleles, two of which that are predicted to be more recessive (H2008, H2022), and two of which that are predicted to be more dominant (H4015, H4035) than H4004n in pollen (Fig. 2). Among the 27 diploid hybrids (F individuals), 21 were carrying at least one copy of the H4004n allele, whereas six did not, so that the latter can be hypothesized to be SI. Accordingly, these six individuals expressed a functional *SCR* allele from *C. grandiflora*, either H2008, H2022 or H4015 (Table 1). Among the 21 homoploid hybrids carrying the H4004n allele, three individuals carried H4015, assumed to be dominant over H4004n. Hence, we can hypothesize that these three individuals are also SI, whereas the 18 remaining individuals are expected to be SC. Accordingly, these three individuals expressed the H4015 functional allele from *C. grandiflora*, thus confirming that H4015 is indeed dominant over H4004n. The remaining 18 individuals expressed predominantly the H4004n allele, with two exceptions: individuals F-5-6 and F-10-4 carried H2022 and H4004n but expression of the former was higher than that of the latter. All seven tetraploidized hybrids (Sh individuals) carried H4004n and the recessive H2022 allele, and are thus hypothesized to be SC. Again, *SCR* results confirm that these individuals expressed the H4004n allele only. Finally, the 19 hybrids between tetraploid individuals of *C. grandiflora* and *C. orientalis* (Sd individuals) were all found to carry H4004n, presumably inherited from their *C. orientalis* parent. Nine of them carried at least one of the more dominant *C. grandiflora* alleles H4015 or H4035, and can be assumed to be SI, whereas the other ten individuals received a more recessive *S*-allele from *C. grandiflora*, and are expected to be SC. These predictions were confirmed by *SCR* expression results (Table 1). Overall, in these hybrid individuals, the asymmetric expression of *SCR* sequences was strong, as observed above in *C. grandiflora* individuals, but the range of relative expression was wider (relative expression of the most dominant *SCR* allele ranging from 0.638 to 1.0) including in diploid hybrids, so this is not caused by polyploidy.

S-haplotypes phylogeny based on full SRK exon1 sequences.
In order to understand which *S*-haplotype specificities are shared within and among Arabidopsis and Capsella genera, 23 *SRK* (exon 1) nucleotide sequences from Arabidopsis and Capsella genera were aligned with Muscle v.3.5 (Edgar 2004) using the default strategy and manually checked with Seaview v.5 (Gouy et al. 2021). *ARK3* and *SRK* genes are paralogous therefore an *ARK3* sequence of *Capsella rubella* (Cru) was used as outgroup. On the alignment, Gblocks version 0.91b (Castresana 2000) was applied to remove poorly aligned regions. This resulted in a 1236 nucleotide dataset. The best fitting model under the ML criterion was selected with ModelTest-ng v.0.1.6 (Darriba et al. 2020). The phylogenetic reconstruction was performed by maximum likelihood (ML) using PhyML version 3.0 (Guindon et al. 2010) with the best of SPR and NNI moves on a BioNJ starting trees under the TPM3uf+I+G4 model. Node stability was estimated by 100 non-parametric bootstrap replicates.

Cited in Supplementary Information

Bachmann, J.A., Tedder, A., Fracassetti, M., Steige, K.A., Lafon-Placette, C., Köhler, C., et al. (2021) On the origin of the widespread self-compatible allotetraploid Capsella bursa-pastoris (Brassicaceae). Heredity, 127, 124–134.

Bachmann, J.A., Tedder, A., Laenen, B., Fracassetti, M., Désamoré, A., Lafon-Placette, C., et al. (2019) Genetic basis and timing of a major mating system shift in Capsella. New Phytologist, 224, 505–517.
Billiard, S., Castric, V. & Vekemans, X. (2007) A General Model to Explore Complex Dominance Patterns in Plant Sporophytic Self-Incompatibility Systems. Genetics, 175, 1351.
Charlesworth, D., Bartolome, C., Schierup, M.H. & Mable B.K. (2003) Haplotype Structure of the Stigmatic Self-Incompatibility Gene in Natural Populations of Arabidopsis lyrata. Molecular Biology and Evolution, 20, 1741–1753.
Castresana, J. (2000) Selection of conserved blocks from multiple alignments for their use in phylogenetic analysis. Molecular Biology and Evolution, 17, 540-552.
Castric, V., Bechsgaard, J., Schierup, M.H. & Vekemans, X. (2008) Repeated Adaptive Introgression at a Gene under Multiallelic Balancing Selection. PLOS Genetics, 4, e1000168.
Castric, V. & Vekemans, X. (2007) Evolution under strong balancing selection: How many codons determine specificity at the female self-incompatibility gene SRK in Brassicaceae? BMC Evolutionary Biology, 7, 1–15.
Darriba, D., Posada, P., Kozlov, A.M., Stamatakis, A., Morel, B. & Flouri, T. (2020) ModelTest-NG: A New and Scalable Tool for the Selection of DNA and Protein Evolutionary Models. Molecular Biology and Evolution, 37, 291–294.
Duan, T., Sicard, A., Glémin, S. & Lascoux, M. (2023) Expression pattern of resynthesized allotetraploid Capsella is determined by hybridization, not whole-genome duplication. New Phytologist, 237, 339–353.
Durand, E., Méheust, R., Soucaze, M., Goubet, P.M., Gallina, S., Poux, C., et al. (2014) Dominance hierarchy arising from the evolution of a complex small RNA regulatory network. Science, 346, 1200–1205.
Edgar, R.C. (2004) MUSCLE: Multiple Sequence Alignment with High Accuracy and High Throughput. Nucleic Acids Research, 32, 1792-7.
Genete, M., Castric, V. & Vekemans, X. (2020) Genotyping and De Novo Discovery of Allelic Variants at the Brassicaceae Self-Incompatibility Locus from Short-Read Sequencing Data. Molecular Biology and Evolution, 37, 1193–1201.
Goubet, P.M., Bergès, H., Bellec, A., Prat, E., Helmstetter, N., Mangenot, S., et al. (2012) Contrasted Patterns of Molecular Evolution in Dominant and Recessive Self-Incompatibility Haplotypes in Arabidopsis. PLOS Genetics, 8, e1002495.
Gouy, M., Tanier, E., Comte, N. & Parsons, D.P. (2021) Seaview Version 5: A Multiplatform Software for Multiple Sequence Alignment, Molecular Phylogenetic Analyses, and Tree Reconciliation in Methods in molecular biology (Clifton, N.J. editor) 2231, 241-260.
Guindon, S., Dufayard, J.F., Lefort, V., Anisimova, M., Hordijk, W. & Gascuel, O. (2010) New Algorithms and Methods to Estimate Maximum-Likelihood Phylogenies: Assessing the Performance of PhyML 3.0. Systematic Biology, 59, 307-321.
Guo, Y.L., Bechsgaard, J.S., Slotte, T., Neuffer, B., Lascoux, M., Weigel, D., et al. (2009) Recent speciation of Capsella rubella from Capsella grandiflora, associated with loss of self-incompatibility and an extreme bottleneck. Proceedings of the National Academy of Sciences of the United States of America, 106, 5246–5251.
Guo, Y.L., Zhao, X., Lanz, C. & Weigel, D. (2011) Evolution of the *S*-Locus Region in Arabidopsis Relatives. Plant Physiology, 157, 937–946.
Josephs, E.B., Lee, Y.W., Stinchcombe, J.R. & Wright, S.I. (2015) Association mapping reveals the role of purifying selection in the maintenance of genomic variation in gene expression. Proceedings of the National Academy of Sciences of the United States of America, 112, 15390–15395.
Kasianov, A.S., Klepikova, A.V., Kulakovskiy, I.V., Gerasimov, E.S., Fedotova, A.V., Besedina, E.G., et al. (2017) High-quality genome assembly of Capsella bursa-pastoris reveals asymmetry of regulatory elements at early stages of polyploid genome evolution. The Plant Journal, 91, 278–291.
Kryvokhyzha, D., Milesi, P., Duan, T., Orsucci, M., Wright, S.I., Glémin, S., et al. (2019) Towards the new normal: Transcriptomic convergence and genomic legacy of the two subgenomes of an allopolyploid weed (Capsella bursa-pastoris). PLoS Genetics, 15, e1008131.

Mable, B.K., Brysting, A.K., Jørgensen, M.H., Carbonell, A.K.Z., Kiefer, C., Ruiz-Duarte, P., et al. (2018) Adding complexity to complexity: Gene family evolution in polyploids. Frontiers in Ecology and Evolution, 6, 114.
Nasrallah, J.B., Liu, P., Sherman-Broyles, S., Schmidt, R. & Nasrallah, M.E. (2007) Epigenetic Mechanisms for Breakdown of Self-Incompatibility in Interspecific Hybrids. Genetics, 175, 1965.
Neuffer, B., Bechsgaard, J., Paetsch, M., Titel, C., Wesse, C., Bona, E., et al. (2023) *S*-alleles and mating system in natural populations of Capsella grandiflora (Brassicaceae) and its congeneric relatives. Flora, 299, 152206.
Paetsch, M., Mayland-Quellhorst, S. & Neuffer, B. (2006) Evolution of the self-incompatibility system in the Brassicaceae: identification of *S*-locus receptor kinase (SRK) in self-incompatible Capsella grandiflora. Heredity 2006 97:4, 97, 283–290.
Prigoda, N.L., Nassuth, A. & Mable, B.K. (2005) Phenotypic and Genotypic Expression of Self-incompatibility Haplotypes in Arabidopsis lyrata Suggests Unique Origin of Alleles in Different Dominance Classes. Molecular Biology and Evolution, 22, 1609–1620.
Schierup, M.H., Vekemans, X. & Christiansen, F.B. (1997) Evolutionary Dynamics of Sporophytic Self-Incompatibility Alleles in Plants. Genetics, 147, 835–846.
Takou, M., Hämälä, T., Koch, E.M., Steige, K.A., Dittberner, H., Yant, L., et al. (2021) Maintenance of Adaptive Dynamics and No Detectable Load in a Range-Edge Outcrossing Plant Population. Molecular Biology and Evolution, 38, 1820–1836.

Supplemental Tables

Table S1. Fully resolved *S*-locus genotypes of 176 individuals from population Cg-9 of *Capsella grandiflora*, as obtained by the NGSgenotyp pipeline on whole-genome resequencing data from Josephs *et al.* (2015). Allele sequences are identified according to a Brassicaceae functional sequence group nomenclature, as determined based on sequence similarity with alleles from related species of *Capsella* and *Arabidopsis* genera (see Table S2 for correspondence with Capsella-specific *S*-allele IDs). The presence of putative polymorphic paralogous sequences (H0002=CgrSRK51, H0003=CgrSRK63, H0011=CgrSRK01, H0012=CgrSRK09, H0012=CgrSRK06) is also reported.

Individual	SRA run	allele 1	allele 2	paralogous sequences
1	SRR2065265	H2015	H3001	
2	SRR2070905	H1001	H4038	
3	SRR2070909	H1001	H3001	
4	SRR2065291	H3001	H2008	H0002,H0011,H0012
5	SRR2065298	H1001	H4026	
6	SRR2070922	H4030	H4040	
7	SRR2070923	H1001	H3029	H0013
9	SRR2065335	H3014	H2008	
10	SRR2065181	H1001	H3007	H0012
11	SRR2065191	H4010	H4028	H0011,H0012
13	SRR2065208	H4020	H2021	
14	SRR2065217	H4010	H3016	
15	SRR2065226	H4004	H3005	
16	SRR2065235	H4019	H4011	
17	SRR2065247	H3005	H2021	
18	SRR2065256	H4020	H1001	
19	SRR2065263	H3013	H2017	H0003
20	SRR2065270	H4001	H4028	
23	SRR2065271	H3012	H3022	
24	SRR2065272	H1001	H2002	H0012
25	SRR2065273	H1001	H2021	
26	SRR2065274	H2015	H1001	
27	SRR2065275	H1001	H4015	H0013

28	SRR2065276	H4004	H3002	
29	SRR2065277	H4008	H3008	H0012
30	SRR2065278	H3022	H4006	
31	SRR2065279	H2006	H4001	
32	SRR2070910	H3015	H2017	
33	SRR2065280	H4024	H3005	H0012
35	SRR2065282	H1001	H2008	
36	SRR2065283	H2010	H2019	
37	SRR2070911	H3017	H4045	
38	SRR2065284	H3023	H2020	
39	SRR2065285	H2005	H2010	
41	SRR2065286	H1001	H4004	
42	SRR2070912	H2002	H4017	H0012
43	SRR2065287	H3013	H4004	H0012
44	SRR2065288	H1001	H3005	
45	SRR2070913	H1001	H4010	
46	SRR2070914	H1001	H2017	
47	SRR2070915	H2002	H4017	
47	SRR2065289	H1001	H4002	
48	SRR2070916	H2011	H4011	
49	SRR2065290	H3013	H3002	
50	SRR2065292	H1001	H1001	
51	SRR2065293	H1001	H1001	H0012
52	SRR2065294	H1001	H1001	
53	SRR2070918	H1001	H1001	
54	SRR2065295	H3029	H3029	H0012
55	SRR2065296	H4040	H4046	
58	SRR2065297	H4027	H3023	
59	SRR2070919	H1001	H4040	
60	SRR2070920	H2010	H4028	
61	SRR2065299	H1001	H3016	H0003
63	SRR2065300	H1001	H2018	
64	SRR2065301	H2017	H2021	
65	SRR2065303	H1001	H3029	
66	SRR2065304	H2012	H3029	
67	SRR2065305	H1001	H1001	

70	SRR2065306	H4040	H3006	
71	SRR2065307	H1001	H3006	
72	SRR2065308	H3005	H4025	
74	SRR2065309	H3012	H4037	
75	SRR2065310	H1001	H2012	
76	SRR2065311	H1001	H3015	H0013
78	SRR2065312	H2015	H3014	
79	SRR2065313	H2008	H2018	
80	SRR2065315	H2002	H3001	
81	SRR2065316	H1001	H3001	
82	SRR2065317	H1001	H4024	
83	SRR2065318	H1001	H3005	
85	SRR2065319	H1001	H3022	
86	SRR2065320	H2015	H3005	
88	SRR2070924	H1001	H4009	
89	SRR2065321	H2017	H2021	H0002
90	SRR2065323	H3015	H3016	
91	SRR2065324	H4007	H3023	H0002
91	SRR2070925	H2006	H4010	
92	SRR2065325	H3016	H4038	
93	SRR2065327	H3017	H3023	
94	SRR2065328	H1001	H1001	
95	SRR2065329	H1001	H2022	
96	SRR2065330	H1001	H4028	
97	SRR2065332	H1001	H3015	H0002
98	SRR2065333	H4010	H4028	H0011
99	SRR2065334	H3005	H4042	H0011
101	SRR2065172	H2008	H4030	H0013
103	SRR2065174	H4001	H4003	H0003
105	SRR2065175	H4027	H3029	
106	SRR2065176	H1001	H4035	
107	SRR2065177	H3017	H4011	
108	SRR2065179	H4011	H4030	H0011, H0013
109	SRR2065180	H1001	H1001	
110	SRR2065182	H1001	H1001	
111	SRR2065184	H4010	H4038	H0002

112	SRR2065185	H1001	H2017	
113	SRR2065186	H2002	H4011	
115	SRR2065188	H4009	H4047	H0012
116	SRR2070888	H3015	H4006	
117	SRR2065189	H1001	H1001	H0013
118	SRR2065190	H3005	H4001	
119	SRR2070889	H2017	H2010	H0012
120	SRR2070890	H2010	H2020	H0003
121	SRR2065192	H2022	H4046	
123	SRR2065193	H1001	H2006	
124	SRR2065195	H4020	H3006	
125	SRR2065196	H3002	H4035	H0013
126	SRR2065197	H1001	H4028	
128	SRR2065198	H3010	H4036	
129	SRR2065199	H4004	H3007	
130	SRR2070892	H3001	H2022	
131	SRR2065200	H2015	H2020	
132	SRR2065201	H1001	H3014	
133	SRR2065202	H1001	H3003	
135	SRR2065203	H1001	H4034	
136	SRR2065204	H3015	H4047	H0013
137	SRR2065205	H4017	H2008	H0013
138	SRR2065206	H4024	H2020	H0013
139	SRR2065207	H1001	H3001	H0002
140	SRR2065209	H3003	H3013	
141	SRR2065210	H1001	H4035	H0012
142	SRR2065211	H1001	H1001	H0003,H0012, H0013
143	SRR2065212	H2011	H2023	
144	SRR2065213	H2012	H4017	
145	SRR2070893	H4013	H4030	
146	SRR2065214	H3001	H2010	
147	SRR2065215	H3002	H3029	
148	SRR2065216	H3010	H2020	
149	SRR2070894	H1001	H4037	H0003
151	SRR2065218	H3016	H4002	
152	SRR2065219	H4001	H4045	

153	SRR2065220	H2010	H4034	
154	SRR2065221	H1001	H2022	
155	SRR2065222	H3003	H3014	
156	SRR2065223	H4036	H4003	
157	SRR2065224	H1001	H4029	
158	SRR2065225	H4017	H4006	H0002
160	SRR2065227	H2002	H4035	H0012
161	SRR2065228	H1001	H1001	
162	SRR2065229	H1001	H3014	H0003,H0011, H0012
163	SRR2065230	H1001	H4014	H0002
165	SRR2065232	H3011	H3022	
166	SRR2070895	H4020	H1001	H0011, H0012
167	SRR2065234	H1001	H2012	
168	SRR2070896	H2012	H3019	
170	SRR2065237	H4027	H4015	
172	SRR2070897	H3017	H2018	
173	SRR2065239	H2012	H4003	H0002,H0012
174	SRR2065241	H4002	H4013	
175	SRR2065242	H1001	H4018	H0012, H0013
176	SRR2065244	H2023	H4028	
177	SRR2065245	H1001	H2002	
178	SRR2065246	H4023	H4029	
179	SRR2070898	H2010	H2010	H0012
180	SRR2070900	H1001	H2020	
181	SRR2065248	H3003	H4030	H0003,H0012
182	SRR2065250	H1001	H4039	
183	SRR2065251	H4020	H3029	
184	SRR2065252	H1001	H4025	
186	SRR2065254	H1001	H4001	H0003
187	SRR2065255	H2013	H4001	
189	SRR2070901	H1001	H2022	
190	SRR2065257	H2015	H2010	
192	SRR2065258	H1001	H3011	
193	SRR2065259	H1001	H4002	
195	SRR2065260	H2002	H4001	
197	SRR2065261	H1001	H3008	

198	SRR2065262	H1001	H3003	H0003
199	SRR2070904	H1001	H2005	
200	SRR2065266	H1001	H1001	
202	SRR2065267	H4001	H2020	H0012
203	SRR2065268	H4027	H3010	
204	SRR2065269	H1001	H4038	
207	SRR2070906	H2005	H4026	
208	SRR2070907	H1001	H4003	
209	SRR2070908	H4004	H4003	H0012

Table S2. List of the 74 *SRK* alleles of *C. grandiflora* identified within population Cg-9. References and Genbank accession numbers of previously published allele sequences are given, as well the source individual from population Cg-9 used to assemble a full sequence of exon 1 using the NGSgenotyp pipeline (available from file S1). References to five putative paralogous sequences are also given.

Allele ID (functional group)	*Capsella* specific allele ID	Genbank	Original source	Individual used to extract full sequence	*A. halleri* homolog	*A. lyrata* homolog	Completeness of exon 1 sequence
H1001	CgrSRK03	DQ530639	Paetsch et al. 2006	75_Contig_N1	AhSRK01	AlSRK01	complete
H2002	CgrSRK10	MT592932	Bechsgaard & Paetsch 2020	113_Contig_N5			complete
H2005	CgrSRK08	MT592926	Bechsgaard & Paetsch 2020	199_Contig_N7	AhSRK23		complete
H2006	CgrSRK24	FJ649962	Guo et al. 2009	123_Contig_N4	AhSRK28	AlSRK28	complete
H2008	CgrSRK38	FJ649960	Guo et al. 2009	101_Contig_N5		AlSRK06	complete
H2010	CgrSRK39	MT592972	Bechsgaard & Paetsch 2020	119_Contig_N4	AhSRK69		complete
H2011	CgrSRK34	FJ613330	Paetsch et al. 2006	143_Contig_N1			complete
H2013	CgrSRK23	MT592948	Bechsgaard & Paetsch 2020	/			partial
H2015	CgrSRK04	DQ530640	Paetsch et al. 2006	1_Contig_N3			complete
H2017	CgrSRK25	MT592950	Bechsgaard & Paetsch 2020	112_Contig_N3			complete
H2018	CgrSRK45	MT592980	Bechsgaard & Paetsch 2020	63_Contig_N5	AhSRK68		complete
H2019	CgrSRKH2019	/	This study	36_Contig_N3			complete
H2020	CgrSRK49	MT592986	Bechsgaard & Paetsch 2020	138_Contig_N5			complete
H2021	CgrSRK05	DQ530641	Paetsch et al. 2006	64_Contig_N3			complete
H2022	CgrSRK27	MT592955	Bechsgaard & Paetsch 2020	130_Contig_N6			complete
H2023	CgrSRKH2023	/	This study	143_Contig_N2			complete
H2024	CgrSRK40	MT592976	Bechsgaard & Paetsch 2020	75_Contig_N2			complete
H3001	CgrSRK18	FJ649953	Guo et al. 2009	130_Contig_N4	AhSRK02	AlSRK17	complete
H3002	CgrSRK17	LR596609	Bachmann et al. 2019	/	AhSRK04	AlSRK37	complete
H3003	CgrSRK12	MT592934	Bechsgaard & Paetsch 2020	140_Contig_N2	AhSRK06	AlSRK21	complete
H3005	CgrSRK32	MT592960	Bechsgaard & Paetsch 2020	86_Contig_N1	AhSRK14		complete
H3006	CgrSRKH3006	/	This study	70_Contig_N5	AhSRK17		complete
H3007	CgrSRKH3007	/	This study	10_Contig_N7	AhSRK25		complete

H3008	CgrSRKH3008	/	This study	29_Contig_N3	AhSRK29	AlSRK13	complete
H3010	CgrSRK46	MT592982	Bechsgaard & Paetsch 2020	203_Contig_N7	AlSRK05		complete
H3011	CgrSRK53	MT592992	Bechsgaard & Paetsch 2020	192_Contig_N6	AhSRK57	AlSRK25	complete
H3012	CgrSRK11	FJ649956	Guo et al. 2009	23_Contig_N4	AhSRK60		complete
H3013	CgrSRK13	MT592935	Bechsgaard & Paetsch 2020	140_Contig_N3			complete
H3014	CgrSRK19	MT592944	Bechsgaard & Paetsch 2020	132_Contig_N6	AhSRK58		complete
H3015	CgrSRK20	MT592945	Bechsgaard & Paetsch 2020	116_Contig_N4	AhSRK70	AlSRK70	complete
H3016	CgrSRK30	MT592958	Bechsgaard & Paetsch 2020	14_Contig_N1			complete
H3017	CgrSRK33	MT592961	Bechsgaard & Paetsch 2020	37_Contig_N1			complete
H3019	CgrSRK43	MT592979	Bechsgaard & Paetsch 2020	168_Contig_N4			complete
H3022	CgrSRKH3022	/	This study	165_Contig_N5	AhSRK63	AlSRK41	complete
H3023	CgrSRKH3023	/	This study	58_Contig_N5		AlSRK48	complete
H3029	CgrSRKH3029	/	This study	147_Contig_N6			complete
H3030	CgrSRK07	EF530735	Nasrallah et al. 2007	85_Contig_N2			complete
H4001	CgrSRK41	MT592977	Bechsgaard & Paetsch 2020	103_Contig_N2	AhSRK05	AlSRK34	complete
H4002	CgrSRK60	MT593000	Bechsgaard & Paetsch 2020	47_Contig_N2	AhSRK07	AlSRK72	complete
H4003	CgrSRKH4003	/	This study	103_Contig_N4	AhSRK11	AlSRK11	complete
H4004	CgrSRK16*	LR596551	Bachmann et al. 2019	/	AhSRK12	AlSRK42	complete
H4006	CgrSRKH4006	/	This study	158_Contig_N6	AhSRK15	AlSRK71	complete
H4007	CgrSRK48	MT592985	Bechsgaard & Paetsch 2020	91_Contig_N5	AhSRK16	AlSRK31	complete
H4008	CgrSRK15	MT592939	Bechsgaard & Paetsch 2020	29_Contig_N4	AhSRK18	AlSRK39	complete
H4009	CgrSRK58	MT592998	Bechsgaard & Paetsch 2020	115_Contig_N4	AhSRK20	AlSRK69	complete
H4010	CgrSRK26	MT592954	Bechsgaard & Paetsch 2020	111_Contig_N3	AhSRK21	AlSRK15	complete
H4011	CgrSRK61	MT593001	Bechsgaard & Paetsch 2020	100_Contig_N5	AhSRK22	AlSRK46	complete
H4012	CgrSRK29	MT592957	Bechsgaard & Paetsch 2020	45_Contig_N7	AhSRK24	AlSRK20	partial
H4013	CgrSRKH4013	/	This study	174_Contig_N3	AhSRK26	AlSRK22	complete
H4014	CgrSRKH4014	/	This study	163_Contig_N5	AhSRK32	AlSRK68	complete
H4015	CgrSRK62	MT593002	Bechsgaard & Paetsch 2020	170_Contig_N2	AhSRK34	AlSRK09	complete
H4017	CgrSRKH4017	/	This study	42_Contig_N2	AhSRK36	AlSRK36	complete
H4018	CgrSRKH4018	/	This study	175_Contig_N4	AhSRK37	AlSRK27	complete
H4019	CgrSRK35	FJ613331	Paetsch et al. 2006	16_Contig_N3	AhSRK38	AlSRK65	complete
H4020	CgrSRK02	DQ530638	Paetsch et al. 2006	124_Contig_N7	AhSRK39	AlSRK35	complete
H4023	CgrSRK14	FJ649958	Guo et al. 2009	178_Contig_N2	AhSRK43	AlSRK43	complete

H4024	CgrSRK21	MT592946	Bechsgaard & Paetsch 2020	138_Contig_N4	AhSRK47	AlSRK63	complete
H4025	CgrSRKH4025	/	This study	72_Contig_N2	AhSRK71	AlSRK04	complete
H4026	CgrSRK31	MT592959	Bechsgaard & Paetsch 2020	5_Contig_N3	AhSRK42	AlSRK23	complete
H4027	CgrSRK37	FJ649951	Guo et al. 2009	58_Contig_N1`	AhSRK62	AlSRK30	complete
H4028	CgrSRKH4028	/	This study	11_Contig_N2	AhSRK51	AlSRK33	complete
H4029	CgrSRKH4029	/	This study	157_Contig_N1	AhSRK64	AlSRK38	complete
H4030	CgrSRKH4030	/	This study	145_Contig_N6	AhSRK50	AlSRK50	complete
H4034	CgrSRK59	MT592999	Bechsgaard & Paetsch 2020	153_Contig_N6	AhSRK54	AlSRK73	complete
H4035	CgrSRK22	MT592947	Bechsgaard & Paetsch 2020	106_Contig_N7	AhSRK31	AlSRK19	complete
H4036	CgrSRK54	MT592994	Bechsgaard & Paetsch 2020	128_Contig_N3	AhSRK55	AlSRK12	complete
H4037	CgrSRKH4037	/	This study	74_Contig_N7	AhSRK66	AlSRK45	complete
H4038	CgrSRKH4038	/	This study	111_Contig_N4	AhSRK52	AlSRK44	complete
H4039	CgrSRK57	MT592997	Bechsgaard & Paetsch 2020	182_Contig_N4	AhSRK56	AlSRK61	complete
H4040	CgrSRKH4040	/	This study	6_Contig_N2	AhSRK44	AlSRK62	complete
H4042	CgrSRKH4042	/	This study	99_Contig_N5	AhSRK46		complete
H4045	CgrSRKH4045	/	This study	37_Contig_N5			complete
H4046	CgrSRKH4046	/	This study	121_Contig_N3			complete
H4047	CgrSRKH4047	/	This study	136_Contig_N7			complete
putative paralogous sequences							
H0002	CgrSRK51	MT592989	Bechsgaard & Paetsch 2020	139_Contig_N5		Aly13-2	complete
H0003	CgrSRK63	MW291556	Neuffer et al. 2023	34_Contig_N3		Aly13-7	complete
H0011	CgrSRK01	DQ530637	Paetsch et al. 2006	11_Contig_N1			complete
H0012	CgrSRK09	MT592927	Bechsgaard & Paetsch 2020	119_Contig_N2			complete
H0013	CgrSRK06	DQ530642	Paetsch et al. 2006	101_Contig_N3			complete

*CgSRK16 has been named CgS12 by Bachmann *et al.* (2019) in analogy to its closely trans-specific sequence AhSRK12 from *A. halleri*.

Table S3. *S*-locus genotypes of individuals from *C. grandiflora*, *C. orientalis* and *C. bursa-pastoris*, as inferred from genomic DNA or RNA-seq data from flower buds, leaf or root tissues (based on data from Kryvokhyzha et al. 2019). References and specific notations for *C. grandiflora* alleles are given in Table S2. H4004n is a non-functional allelle present in *C. orientalis* and *C. bursa-pastoris* (Bachmann et al. 2019; Bachmann et al. 2021), derived from the functional H4004 allele from *C. grandiflora*. H4047n is a non-functional allele present in *C. bursa-pastoris*, that has not yet been identified in *C. grandiflora* (Bachmann et al. 2021). The presence of paralogous sequences (H0003, H0011, H0014) is also indicated (references given in Table S2; H0014 is equivalent to CorSRK06 described in Neuffer et al. 2022).

**Species (Geographical group*)**	**Accession ID**	**Genomic DNA**		**RNA flower buds**		**RNA leaf**		**RNA root**	
		allele 1 *(paralogues)*	allele 2 (allele 3)	allele 1	allele 2 (allele 3)	allele 1	allele 2	allele 1	allele 2
C. grandiflora	85.3	H2002 *(H0011)*	H4021	H2002 *(H0011)*	H4021	H2002 *(H0011)*	/	H2002 *(H0011)*	/
C. grandiflora	86.12	H1001 *(H0003)*	H2006	H1001 *(H0003)*	H2006	H2006	/	H2006	/
C. grandiflora	87.26	H1001	H3014	H1001	H3014	H3014	/	H3014	/
C. grandiflora	88.5	H1001	H2017	H1001	H2017	/	/	/	/
C. orientalis	GUB-RUS5	H4004n *(H0014)*	H4004n	H4004n *(H0014)*	H4004n	/ *(H0014)*	/	/ *(H0014)*	/
C. orientalis	PAR-RUS	H4004n *(H0014)*	H4004n	H4004n *(H0014)*	H4004n	/	/	/	/

**Species (Geographical group*)**	**Accession ID**	**Genomic DNA**		**RNA flower buds**		**RNA leaf**		**RNA root**	
C. orientalis	QH-CHIN4	H4004n *(H0014)*	H4004n	H4004n *(H0014)*	H4004n	/ *(H0014)*	/	/	/
C. orientalis	URAL-RUS4	H4004n *(H0014)*	H4004n	H4004n *(H0014)*	H4004n	/	/	/	/
C. bursa-pastoris (ASI)	DL174	H4047n *(H0003)*	H4004n *(H0014)*	H4047n *(H0003)*	H4004n *(H0014)*	/	/	/	/
C. bursa-pastoris (ASI)	JZH152	H4047n *(H0003)*	H4004n *(H0014)*	H4047n *(H0003)*	H4004n *(H0014)*	/	/	/	/
C. bursa-pastoris (ASI)	NJ219	H4047n *(H0003)*	H4004n *(H0014)*	H4047n *(H0003)*	H4004n *(H0014)*	/	/	/	/
C. bursa-pastoris (ASI)	TY118	H4047n *(H0003)*	H4004n *(H0014)*	H4047n *(H0003)*	H4004n *(H0014)*	/	/	/	/
C. bursa-pastoris (CASI)	DUB-RUS9	H4047n *(H0003)*	H2002 *(H0014)*	H4047n *(H0003)*	H2002 *(H0014)*	/	/	H2002	/
C. bursa-pastoris (CASI)	KYRG-3-14	H4047n	H4004n *(H0014)*	H4047n	H4004n *(H0014)*	/	/	/	/
C. bursa-pastoris (CASI)	LAB-RUS-4	H4047n	H4004n (H2002)	H4047n *(H0003)*	H4004n (H2002)	/	/	H2002	/
C. bursa-pastoris (CASI)	TACH-CHIN14	H4047n *(H0003)*	H4004n	H4047n *(H0003)*	H4004n	/	/	/	/
C. bursa-pastoris (EUR)	FR50	H4047n *(H0003)*	H4004n	H4047n *(H0003)*	H4004n	/	/	/	/
C. bursa-pastoris (EUR)	SE33	H4047n *(H0003)*	H4004n	H4047n *(H0003)*	H4004n	/	/	/	/

**Species (Geographical group*)**	**Accession ID**	**Genomic DNA**		**RNA flower buds**		**RNA leaf**		**RNA root**	
C. bursa-pastoris (EUR)	STA4	H4047n *(H0003)*	H4004n	H4047n *(H0003)*	H4004n	/	/	/	/
C. bursa-pastoris (EUR)	STJ2	H4047n *(H0003)*	H4004n *(H0014)*	H4047n *(H0003)*	H4004n *(H0014)*	/	/	/	/
C. bursa-pastoris (ME)	AL87	H4047n *(H0003)*	H4004n *(H0014)*	H4047n *(H0003)*	H4004n *(H0014)*	/	/	/	/
C. bursa-pastoris (ME)	JO56	H4047n *(H0003)*	H4004n *(H0014)*	H4047n *(H0003)*	H4004n *(H0014)*	/	/	/	/
C. bursa-pastoris (ME)	JO59	H4047n *(H0003)*	H4004n *(H0014)*	H4047n *(H0003)*	H4004n *(H0014)*	/	/	/	/
C. bursa-pastoris (ME)	TR73	H4047n *(H0003)*	H4004n *(H0014)*	H4047n *(H0003)*	H4004n *(H0014)*	/	/	/	/

*Geographical groups of *C. bursa-pastoris* : ASI, Asian; CASI, Central Asion; EUR, European; ME, Middle East

Table S4. Relative expression levels of co-existing *S*-alleles in *SRK* and *SCR* in diploid and tetraploid individuals of *C. grandiflora* (cg2 and cg4) and *C. orientalis* (co2 and co4). The predominantly expressed allele in pollen is compared to putative dominance determined by previous knowledge about the dominance classes of the alleles. The difference in expression of the dominant allele is reported as a ratio of mean read depth, obtained by the genotyping pipeline NGSgenotyp.

Individual	ploidy	allele 1	*SRK* read depth	*SCR* read depth	allele 2	*SRK* read depth	*SCR* read depth	allele 3	*SRK* read depth	*SCR* read depth	Putative dominant allele in pollen	Allele expressed predomi-nantly in pollen	Relative SCR expression level of the dominant allele
cg2-1-2-F	2x	H4015	15.12	931.38	H2022	76.60	0.00				H4015	H4015	1.0000
cg2-1-6-F	2x	H4015	13.06	1002.80	H1001	3.39	2.80				H4015	H4015	0.9972
cg2-2-6-F	2x	H4015	7.53	1110.60	H2022	50.05	0.00				H4015	H4015	1.0000
cg2-7-3-F	2x	H4015	22.58	1230.06	H1001	7.29	0.00				H4015	H4015	1.0000
cg2-9-5-F	2x	H2022	94.62	7.60	H1001	5.79	0.00				H2022	H2022	1.0000
cg2-12-3-F	2x	H2022	35.86	3.18	H2008	6.47	2.00				H2008 or H2022*	H2022	0.6139
cg2-14-5-F	2x	H2022	90.21	9.75							H2022	H2022	/[§]
cg4-1-3-F	4x	H4035	20.55	348.30	H4015	10.06	18.50	H2022	22.28	2.60	H4035 or H4015*	H4035	0.9429
cg4-6-4-F	4x	H2022	49.90	3.38	H2008	4.68	0.00				H2008 or H2022*	H2022	1.0000

cg4-7-3-F	4x	H2022	21.03	0.78	H2008	7.60	2.15				H2008 or H2022*	H2008	0.7338
cg4-8-3-F	4x	H4035	35.39	1409.43	H2022	12.85	0.00	H2008	6.41	0.00	H4035	H4035	1.0000
cg4-12-4-F	4x	H4035	7.47	340.44	H2022	9.56	1.00	H2008	4.45	0.00	H4035	H4035	0.9971
cg4-9-2-F	4x	H4035	45.29	687.04	H2008	15.60	0.00				H4035	H4035	1.0000
co2-2-6-F	2x	H4004n	20.56	32.21							H4004n	H4004n	1.0000
co4-1-4-F	4x	H4004n	12.63	25.82							H4004n	H4004n	1.0000

*When two alleles belonging to the same dominance class co-occur, we cannot determine a priori which one is the most dominant, but *SCR* expression data allows us to resolve this uncertainty.

§Undetermined as the individual is homozygous.

Table S5. *S*-locus genotypes and inference of putative self-compatible (SC) or self-incompatible (SI) phenotype of diploid and tetraploid individuals from *C. grandiflora* (*cg2* and *cg4*), *C. orientalis* (*co2* and *co4*) and experimental hybrids (F, Sd, Sh), as inferred from RNA-seq data from flower buds and analysis of *SRK* with the NGSgenotyp pipeline. Uncertainties in the relative number of gene copies in some tetraploid individuals are indicated. The putative self-incompatible (SI) vs self-compatible (SC) phenotype of a given individual is inferred from previous knowledge of relative dominance of *S*-alleles in the pollen and from whether the non-functional allele H4004n from *C. orientalis* is present and putatively dominant in this individual. The presence of paralogous sequences (H0002, H0003, H0011, H0012, H0013, H0014) is also indicated.

Individual	ploidy	allele copy 1	allele copy 2	allele copy 3	allele copy 4	putative paralogous sequences	Putative dominant allele	Putative phenotype
cg2-1-2-F	2x	H4015	H2022			H0011,H0013	H4015	SI
cg2-1-6-F	2x	H4015	H1001			H0011	H4015	SI
cg2-2-6-F	2x	H4015	H2022			H0002,H0011	H4015	SI
cg2-7-3-F	2x	H4015	H1001			H0002,H0011	H4015	SI
cg2-9-5-F	2x	H2022	H1001			H0002,H0012	H2022	SI
cg2-12-3-F	2x	H2008	H2022			H0011	H2008 or H2022	SI
cg2-14-5-F	2x	H2022	H2022			H0011,H0012	H2022	SI
cg4-1-3-Fa	4x	H4035	H4015	H2022	H4035, H4015 or H2022	H0011,H0013	H4035 or H4015	SI
cg4-6-4-Fa	4x	H2022	H2008	H2022 or H2008	H2022 or H2008	H0011	H2008 or H2022	SI
cg4-7-3-Fa	4x	H2022	H2008	H2022 or H2008	H2022 or H2008	H0011	H2008 or H2022	SI
cg4-8-3-Fa	4x	H4035	H2022	H2008	H4035, H2022 or H2008	H0011,H0012	H4035	SI
cg4-12-4-Fa	4x	H4035	H2022	H2008	H4035, H2022 or H2008	H0011	H4035	SI

cg4-9-2-Fa	4x	H4035	H2008	H4035 or H2008	H4035 or H2008	H0002,H0011	H4035	SI
co2-11-1-F	2x	H4004n	H4004n			H00014	H4004n	SC
co2-2-6-F	2x	H4004n	H4004n			H00014	H4004n	SC
co2-4-3-F	2x	H4004n	H4004n			H00014	H4004n	SC
co2-6-4-F	2x	H4004n	H4004n			H00014	H4004n	SC
co2-8-5-F	2x	H4004n	H4004n			H00014	H4004n	SC
co2-9-5-F	2x	H4004n	H4004n			H00014	H4004n	SC
co4-1-4-F	4x	H4004n	H4004n	H4004n	H4004n	H00014	H4004n	SC
co4-3-1-F	4x	H4004n	H4004n	H4004n	H4004n	H00014	H4004n	SC
co4-4-6-F	4x	H4004n	H4004n	H4004n	H4004n	H00014	H4004n	SC
co4-8-3-F	4x	H4004n	H4004n	H4004n	H4004n	H00014	H4004n	SC
co4-9-3-F	4x	H4004n	H4004n	H4004n	H4004n	H00014	H4004n	SC
F-1-3	2x	H4004n	H2022			H0002,H0012,H0014	H4004n	SC
F-1-4	2x	H4004n	H2022			H0002,H0012,H0014	H4004n	SC
F-1-5	2x	H4004n	H4004n			H0012	H4004n	SC
F-1-6	2x	H4004n	H2022			H0002,H0012	H4004n	SC
F-3-1	2x	H4004n	H4004n			H0012,H0014	H4004n	SC
F-3-5	2x	H4004n	H2022			H0012,H0014	H4004n	SC
F-5-1	2x	H4004n	H2022			H0011,H0014	H4004n	SC
F-5-3	2x	H4004n	H2022			H0011,H0014	H4004n	SC
F-5-4	2x	H2022	H2022			H0011,H0014	H2022	SI
F-5-5	2x	H2008	H2022			H0011,H0014	H2008 or H2022	SI
F-5-6	2x	H4004n	H2022			H0014	H4004n	SC
F-8-2	2x	H4015	H4015			H0012,H0014	H4015	SI
F-8-3	2x	H4004n	H4015			H0012,H0014	H4015	SI
F-8-4	2x	H4004n	H4004n			H0012,H0014	H4004n	SC
F-8-5	2x	H4004n	H4015			H0012,H0014	H4015	SI
F-8-6	2x	H4004n	H4015			H0012	H4015	SI
F-9-1	2x	H4004n	H2022			H0002,H0012	H4004n	SC
F-9-2	2x	H4004n	H2022			H0002,H0012	H4004n	SC

F-9-4	2x	H2022	H2022			H0002,H0014	H2022	SI
F-9-5	2x	H4004n	H2022			H0002,H0014	H4004n	SC
F-9-6	2x	H4004n	H2022			H0002,H0012,H0014	H4004n	SC
F-10-1	2x	H4004n	H2022				H4004n	SC
F-10-2	2x	H4004n	H4004n			H0014	H4004n	SC
F-10-3	2x	H2022	H2022			H0014	H2022	SI
F-10-4	2x	H4004n	H2022			H0014	H4004n	SC
F-10-5	2x	H4004n	H4004n			H0014	H4004n	SC
F-10-6	2x	H2022	H2022			H0014	H2022	SI
Sd-1-1	4x	H4004n	H2022	H4004n or H2022	H4004n or H2022	H0011,H0014	H4004n	SC
Sd-2-3	4x	H4004n	H2022	H2008	H4004n ,H2022 or H2008	H0011	H4004n	SC
Sd-4-1	4x	H4004n	H4035	H4004n or H4035	H4004n or H4035	H0012,H0014	H4035	SI
Sd-4-2	4x	H4004n	H4015	H4004n or H4015	H4004n or H4015	H0011,H0012,H0014	H4015	SI
Sd-4-3	4x	H4004n	H4035	H4004n or H4035	H4004n or H4035	H0011,H0014	H4035	SI
Sd-4-5	4x	H4004n	H4035	H4015	H4004n ,H4035 or H4015	H0012,H0014	H4035 or H4015	SI
Sd-4-6	4x	H4004n	H4015	H4004n or H4015	H4004n or H4015	H0011,H0012,H0014	H4015	SI
Sd-6-1	4x	H4004n	H2008	H4004n or H2008	H4004n or H2008	H0014	H4004n	SC
Sd-6-3	4x	H4004n	H2008	H4004n or H2008	H4004n or H2008	H0014	H4004n	SC
Sd-6-4	4x	H4004n	H4035	H2008	H4004n ,H4035 or H2008	H0014	H4035	SI
Sd-6-5	4x	H4004n	H2008	H4004n or H2008	H4004n or H2008	H0014	H4004n	SC
Sd-6-6	4x	H4004n	H4035	H4004n or H4035	H4004n or H4035	H0014	H4035	SI
Sd-7-1	4x	H4004n	H4035	H2008	H4004n ,H4035 or H2008	H0011,H0014	H4035	SI
Sd-7-3	4x	H4004n	H2008	H4004n or H2008	H4004n or H2008	H0011,H0014	H4004n	SC
Sd-7-5	4x	H4004n	H4035	H4004n or H4035	H4004n or H4035	H0014	H4035	SI

Sd-8-1	4x	H4004n	H2008	H4004n or H2008	H4004n or H2008	H0002,H0011,H0014	H4004n	SC
Sd-8-4	4x	H4004n	H2022	H4004n or H2022	H4004n or H2022	H0002,H0012,H0014	H4004n	SC
Sd-8-5	4x	H4004n	H2022	H2008	H4004n ,H2022 or H2008	H0002,H0014	H4004n	SC
Sd-8-6	4x	H4004n	H2022	H4004n or H2022	H4004n or H2022	H0002,H0014	H4004n	SC
Sh-1-2	4x	H4004n	H2022	H4004n or H2022	H4004n or H2022	H0012,H0014	H4004n	SC
Sh-2-5	4x	H4004n	H2022	H4004n or H2022	H4004n or H2022	H0002,H0011,H0014	H4004n	SC
Sh-3-5	4x	H4004n	H2022	H4004n or H2022	H4004n or H2022	H0012,H0014	H4004n	SC
Sh-5-5	4x	H4004n	H2022	H2008	H4004n ,H2022 or H2008	H0011,H0014	H4004n	SC
Sh-7-5	4x	H4004n	H2022	H4004n or H2022	H4004n or H2022	H0011	H4004n	SC
Sh-7-6	4x	H4004n	H2022	H4004n or H2022	H4004n or H2022	H0011,H0014	H4004n	SC
Sh-9-2	4x	H4004n	H2022	H2008	H4004n ,H2022 or H2008	H0011,H0012,H0014	H4004n	SC

Table S6. Prediction of self-compatibility phenotype (self-compatible, SC; or self-incompatible, SI) of diploid and tetraploid hybrid individuals based on results on *SRK* genotype of individuals and previous knowledge about dominance relationships among *S*-alleles, and based on actual dominance of the non-functional allele copy of *C. orientalis* as estimated from relative expression levels of *SCR*. Grey boxes indicate the detection of the presence of an *S*-allele from *SRK* genotyping results. Numbers within boxes correspond to the mean read depth of *SCR* of the corresponding allele. Individual F-5-3 was suspected to be a spontaneous allotetraploid based on its phenotypes.

**Hybrid indiv-idual**	**Ploidy**	**Presence/absence of *SRK* and mean read depth of *SCR* allele:**					***SCR* read depth of H4004n relative to total**	**Predicted SI/SC phenotype based on *SRK* genotype**
		**H2008**	**H2022**	**H4004n**	**H4015**	**H4035**		
F-1-3	2x		5.5	**62.0**			0.919	SC
F-1-4	2x		0.0	**3.4**			1	SC
F-1-5	2x			**2026**			1	SC
F-1-6	2x		2.0	**8.8**			0.815	SC
F-3-1	2x			**21.9**			1	SC
F-3-5	2x		4.6	**114.4**			0.961	SC
F-5-1	2x		0.0	**28.2**			1	SC
F-5-3	2x		2.0	**54.7**			0.965	SC
F-5-4	2x		**2.0**				0	SI
F-5-5	2x		**3.3**				0	SI
F-5-6	2x		**282.5**	42.3			0.13*	SC
F-8-2	2x				**2317.5**		0	SI
F-8-3	2x			6.1	**858.9**		0.007	SI
F-8-4	2x			**74.8**			1	SC
F-8-5	2x			4.9	**1628.8**		0.003	SI
F-8-6	2x			15.2	**1110.3**		0.014	SI
F-9-1	2x		17.5	**38.3**			0.686	SC
F-9-2	2x		2.0	**13.2**			0.868	SC
F-9-4	2x		**6.3**				0	SI
F-9-5	2x		2.9	**41.4**			0.935	SC
F-9-6	2x		2.6	**7.2**			0.735	SC
F-10-1	2x		29.3	**109.0**			0.788	SC

F-10-2	2x			**10.6**			1	SC
F-10-3	2x		**2186.0**				0.000	SI
F-10-4	2x		**66.4**	47.6			0.418*	SC
F-10-5	2x			**134.4**			1.000	SC
F-10-6	2x		**3670.0**				0.000	SI
Sd-1-1	4x		1.9	**72.8**			0.975	SC
Sd-2-3	4x	0.0	2.2	**66.7**			0.968	SC
Sd-4-1	4x			41.0		**724.2**	0.054	SI
Sd-4-2	4x			14.0	**1694.7**		0.008	SI
Sd-4-3	4x			65.1		**518.3**	0.112	SI
Sd-4-5	4x			7.3	55.0	**762.6**	0.009	SI
Sd-4-6	4x			3.7	**1103.0**		0.003	SI
Sd-6-1	4x	26.0		**116.2**			0.817	SC
Sd-6-3	4x	4.3		**43.7**			0.910	SC
Sd-6-4	4x	10.9		40.9		**438.6**	0.083	SI
Sd-6-5	4x	2.9		**20.7**			0.877	SC
Sd-6-6	4x			20.8		**484.1**	0.041	SI
Sd-7-1	4x	0.0		11.0		**516.8**	0.021	SI
Sd-7-3	4x	0.0		**72.0**			1.000	SC
Sd-7-5	4x			10.8		**491.91**	0.021	SI
Sd-8-1	4x	0.0		**67.9**			1.000	SC
Sd-8-4	4x	0.0	0.0	**100.1**			1.000	SC
Sd-8-5	4x	3.9	0.0	**73.8**			0.950	SC
Sd-8-6	4x		1.7	**224.8**			0.992	SC
Sh-1-2	4x		2.4	**58.0**			0.960	SC
Sh-2-5	4x		0.8	**43.5**			0.982	SC
Sh-3-5	4x		5.5	**34.8**			0.864	SC
Sh-5-5	4x		0.8	**127.7**			0.994	SC
Sh-7-5	4x		2.8	**190.5**			0.986	SC
Sh-7-6	4x		1.0	**51.3**			0.981	SC
Sh-9-2	4x		0.0	**31.5**			1.000	SC

* indicates two individuals with reverse patterns of relative expression of SC than those expected based on knowledge of dominance between class II and class IV.

Fig. S1 Crosses and induced whole-genome duplication (WGD) for generating diploid (F) and two types of tetraploid hybrids (Sd and Sh) from diploid *Capsella orientalis* (Co2) and *Capsella grandiflora* (Cg2; Duan et al. 2023). Autotetraploid *Capsella orientalis* (Co4) and *Capsella grandiflora* (Cg4) were created as intermediate steps. The generation numbers of the synthetic polyploids or hybrids are indicated by subscripts, and plant materials used in the present study are highlighted in bold.

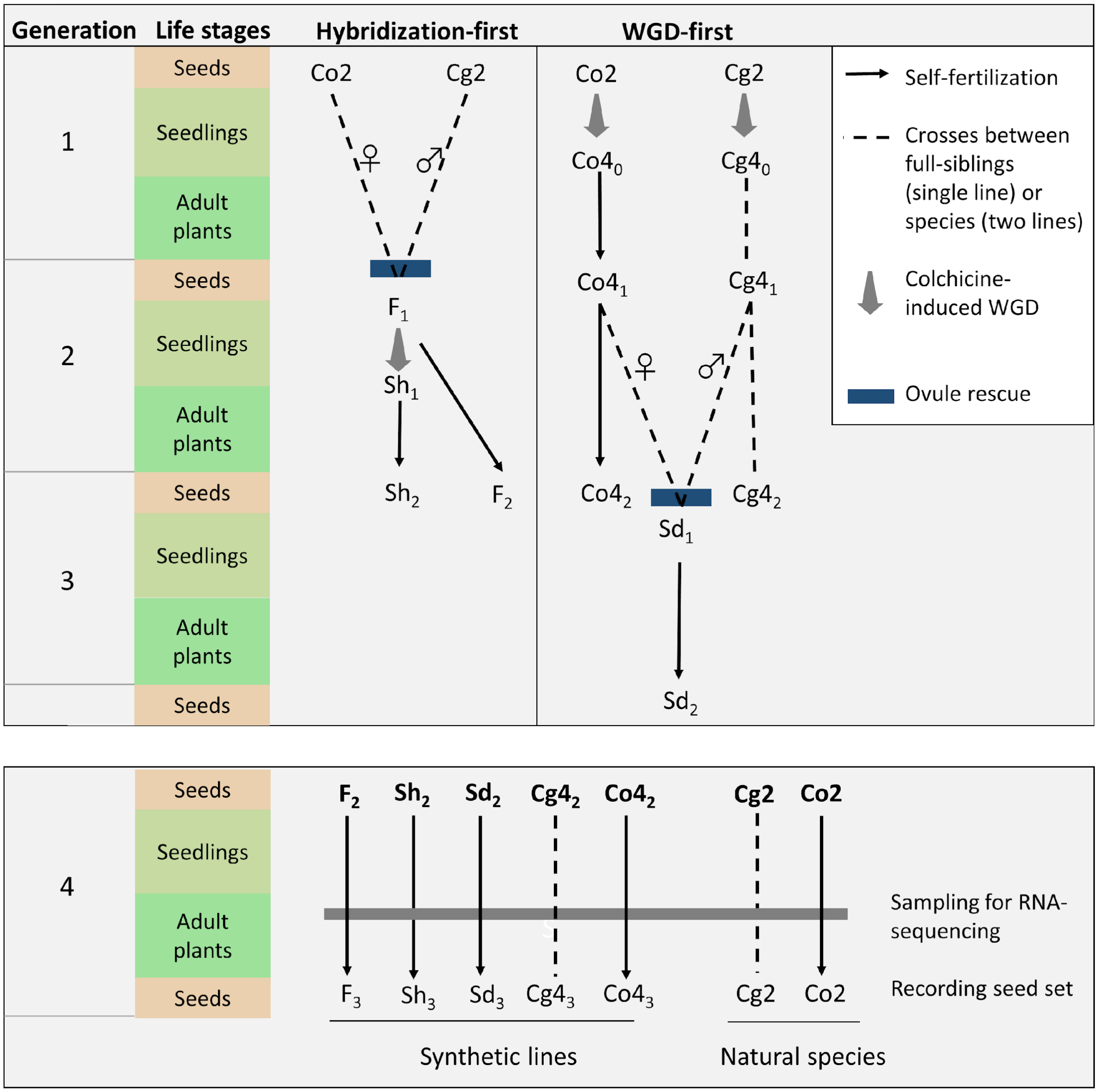

Fig. S2 Phylogeny of *SRK* alleles identified in parents or hybrids between *Capsella grandiflora* (Cgr) and *C. orientalis* (Co). Phylogenetic reconstruction using maximum likelihood under the TPM3uf+I+G4 model implemented by PhyML (see Supplementary Information). We also included sequences identified in *C. bursa-pastoris* (Cbp), and trans-specific alleles from *Arabidopsis halleri* (Ah), *A. lyrata* (Al) or *A. arenosa* (Aar) when available. Functional specificities and dominance classes of the *S*-alleles are indicated. The outgroup is represented by an *ARK3* sequence from *Capsella rubella* (Cru). Bootstrap values are represented by dots on the nodes: black: BP = 100, grey: 95<BP<99, white: BP=89, no dot: BP < 80.

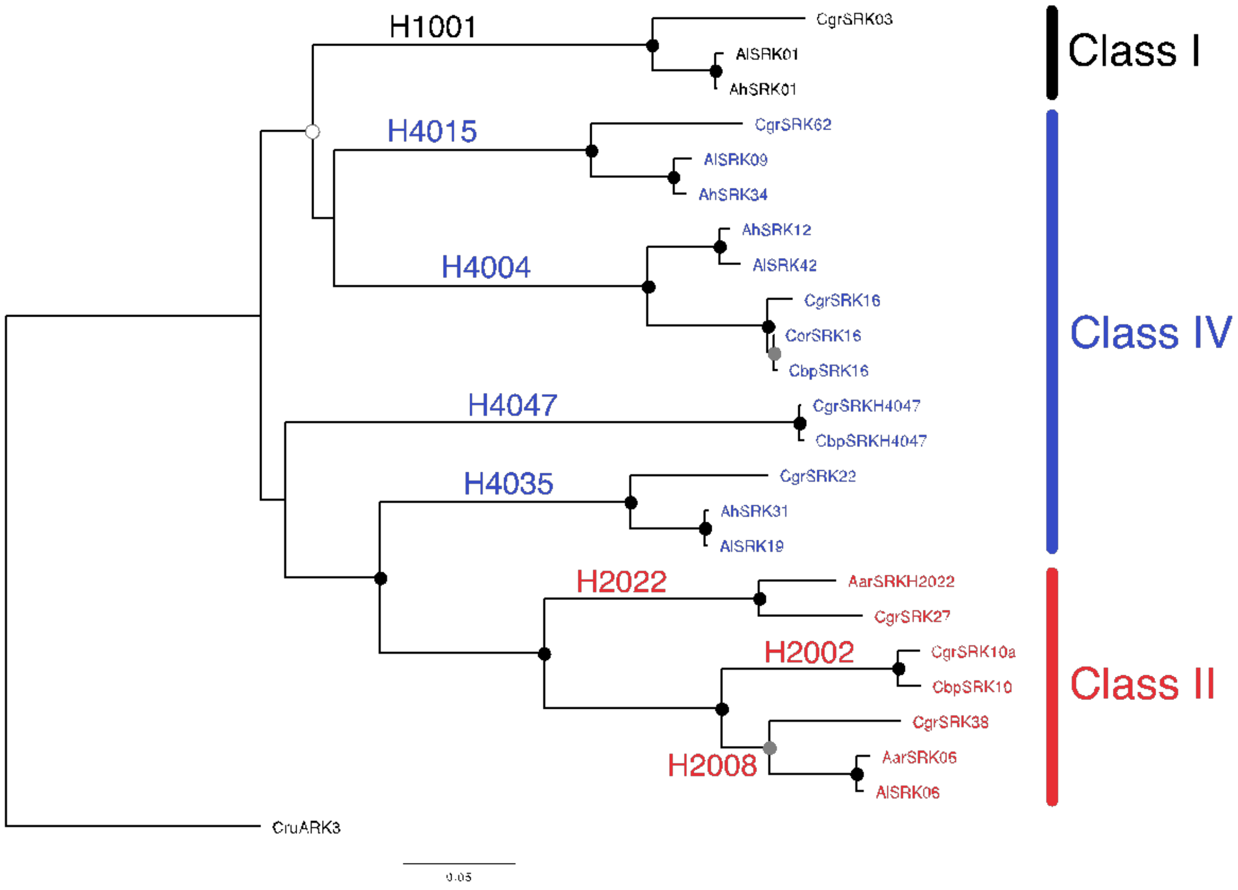

Fig. S3 Observed relative SCR read depth of H4004n relative to all other alleles in diploid and tetraploid hybrids as a function of expected dominance of H4004n (green, H4004n is dominant over *C. grandiflora* allele(s); brown, H4004n is recessive to *C. grandiflora* allele(s)).

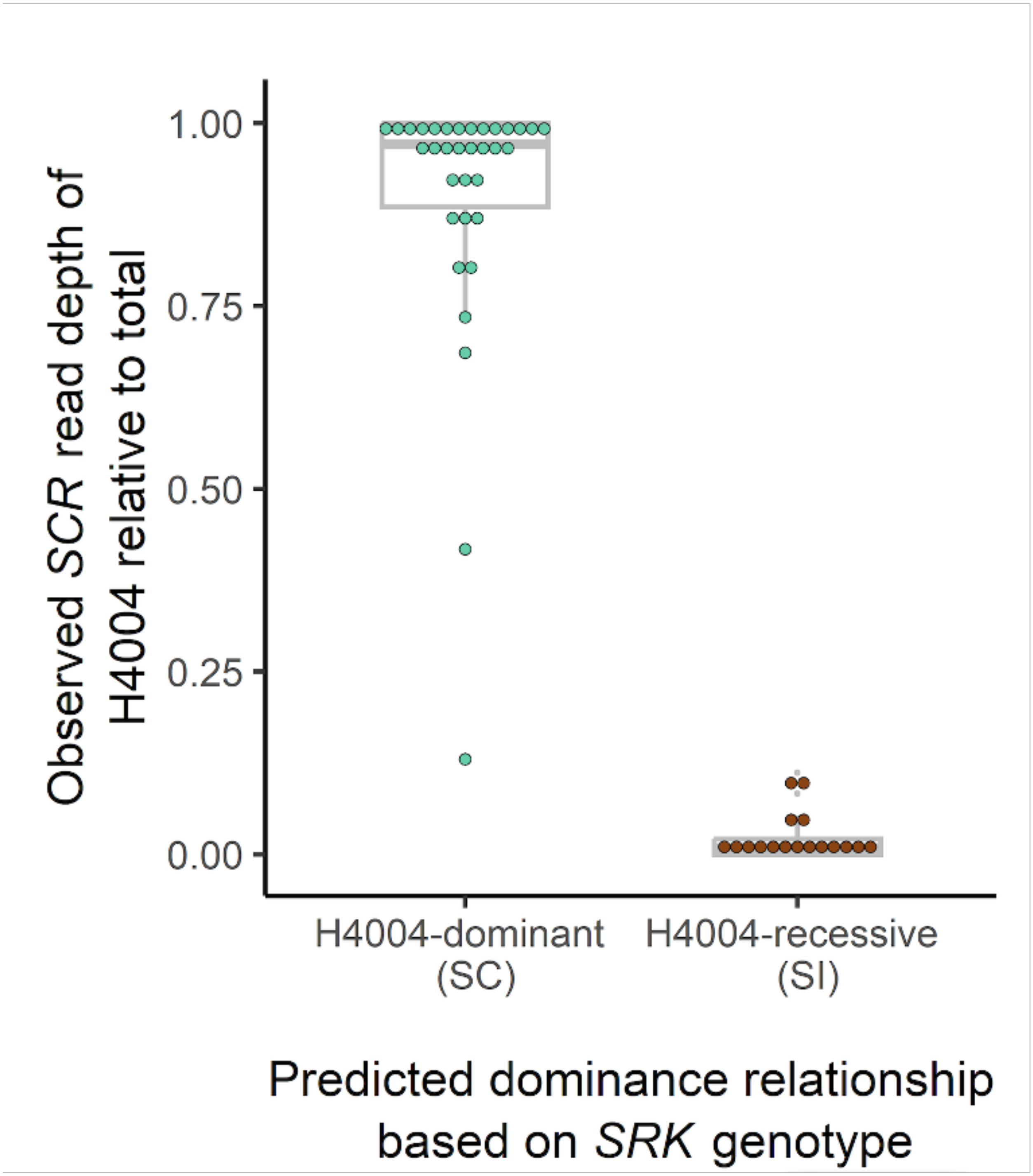

Supplement: qrae011_suppl_Supplementary_Tables_S1-S6_Figures_S1-S4 [file qrae011_suppl_supplementary_tables_s1-s6_figures_s1-s4.pdf]
